# Dynamic functional connectivity is related to cognitive performance of prodromal Lewy body dementia

**DOI:** 10.1007/s00702-026-03148-6

**Published:** 2026-03-31

**Authors:** Martin Gajdoš, Marie Schejbalová, Martin Lamoš, Pavel Říha, Ľubomíra Nováková, Markéta Nečasová, Nela Němcová Elfmarková, Eva Výtvarová, Michal Mikl, Irena Rektorová

**Affiliations:** 1 Brain and Mind Research Program, Central European Institute of Technology (CEITEC), Masaryk University, Kamenice 753/5, 625 00 Brno, Czech Republic; 2 First Department of Neurology, Faculty of Medicine, St. Anne University Hospital, Masaryk University, Brno, Czech Republic; 3 Department of Medical Biophysics, Faculty of Medicine in Hradec Králové, Charles University, Šimkova 870, 500 03 Hradec Králové, Czech Republic

**Keywords:** Dynamic functional connectivity, Resting state fMRI, Dynamic connectivity states, Cognitive domains, Prodromal Lewy body dementia, Fast eigenvector centrality

## Abstract

Brain connectivity dynamics in mild cognitive impairment with Lewy bodies (MCI-LB) is largely unknown. We aimed to identify brain connectivity dynamics related to cognitive performance in drug naïve MCI-LB. Healthy (55 participants, age 67.7 ± 6.4, 33 females) and MCI-LB subjects (30 participants, age 68.5 ± 6.0, 16 females) underwent cognitive testing and fMRI. The fast eigenvector centrality dynamic functional connectivity was employed to detect connectivity states. Mean state duration and occurrence were correlated to cognitive measures. In the MCI-LB group only, the occurrence and mean duration of the state characterized by between-network connectivity of the dorsal attention, sensorimotor, and visual networks correlated with visuospatial function (VSF) domain z-scores (*r* = 0.68, *p* = 0.003, and *r* = -0.68, *p* = 0.003, respectively). The VSF also correlated with the occurrence of the state characterized by intra-network connectivity of the frontoparietal control network (*r* = -0.55, *p* = 0.041). We identified specific mechanisms that seem to facilitate visuospatial performance in MCI-LB.

**Supplementary Information:**

The online version contains supplementary material available at https://doi.org/10.1007/s00702-026-03148-6.

## Introduction

Lewy body dementia (LBD) is the second-most common degenerative dementia after Alzheimer’s disease (AD)(Goodman et al. 2017) which is characterized by core clinical features including parkinsonism, REM sleep behavioral disorder, fluctuating cognition and visual hallucinations, and dementia dominated by dysexecutive syndrome, attentional deficits and visuospatial dysfunction, particularly early during the disease progression, at the stage of mild cognitive impairment (Donaghy et al. 2018; McKeith et al. 2020; van de Beek et al. 2020; Jellinger 2023).

Resting-state functional magnetic resonance imaging (rs-fMRI) is an effective tool for determining changes in brain networks related to LBD (Scamarcia et al. 2021). Using static and dynamic functional connectivity (dFC) (Schumacher et al. 2021), the authors were not able to uncover specific changes in mild cognitive impairment with Lewy bodies (MCI-LB) patients as compared to healthy controls (HC) and mild cognitive impairment due to AD (MCI-AD). However, distinct alterations of the spatiotemporal dynamics of whole-brain resting states were found by using a microstate analysis of EEG data (Lamoš et al. 2021).

It has been already shown that graph properties and dynamic graph properties of functional connectivity as evaluated by eigenvector centrality may reflect pathological changes related to brain neurodegeneration (Lorenzini et al. 2023). In LBD, Schumacher et al. (Schumacher et al. 2019a) reported an increased time in a state of low inter-network connectivity and difficulties in switching into densely connected networks. Furthermore, the authors observed reduction in the temporal variability of global network efficiency, similarly to other works performed in Parkinson’s disease dementia (PDD) (Díez-Cirarda et al. 2018; Fiorenzato et al. 2019), which shares with LBD significant symptomatic and pathological overlap (Aarsland et al. 2009; Friedman 2018; Jellinger and Korczyn 2018).

Fast eigenvector centrality (FEC) is a whole-brain, voxel-wise approach designed to detect dominant patterns of fluctuations (Preti and Van De Ville 2016). By operating at the voxel level, FEC obviates the need for subjective parcellation schemes, which influence observed connectivity dynamics. The identified representative dominant patterns (RDPs) reflect established large-scale resting-state networks (RSNs), including the default mode (DMN), fronto-parietal (FPN), salience, and attention networks (Shirer et al. 2012). Given that RSN disruptions are present in both LBD(Schumacher et al. 2019a; Matar et al. 2022; Firbank et al. 2024; Onofrj et al. 2026) and PD (Onu et al. 2015; Caspers et al. 2021). Using eigenvector centrality on selected clusters, Lorenzini et al. reported reduction of functional integration between RSN in the early stages of Alzheimer’s disease (AD) which further promoted cognitive deterioration (Lorenzini et al. 2023). FEC extends this metric with whole brain and parcellation independent methodology that capture dynamic network-level connectivity in spatially unconstrained manner. Therefore, it offers a promising framework for investigating the prodromal stages of these disorders, such as MCI-LB. We employed rs-fMRI and the FEC to uncover dFC states and their relation to early cognitive deficits in drug-naïve MCI-LB patients. We aimed to enhance our understanding of the role of the brain’s (mal)adaption and compensation related to brain pathology and its impact on cognitive performance, particularly on the cognitive domains that are known to be impaired in the pre-dementia stage, including attention, executive and visuospatial functions. The rationale for using dFC was to assess dynamics of brain network patterns that may be well suited to reflect fluctuating cognition and lapses of attention characteristic of LBD and its prodromal stages. We hypothesized alterations in dynamics of the RSN involved in the LBD development and progression (Chabran et al. 2020), including the sensorimotor network (SMN), dorsal attention network (DAN), DMN, visual network (VN), and frontoparietal control network (FPCN), particularly in their relation to cognitive performance in the cognitive domains of interest.

## Methods

### Participants

Altogether, 37 participants diagnosed with MCI-LB and 65 healthy controls (HC) entered the study. A participant was assigned to the MCI-LB group based on a neuropsychological examination and the simultaneous occurrence of two or more core clinical symptoms of LBD as described previously (McKeith et al. 2020; Novakova et al. 2024; Železníková et al. 2024). All MCI-LB participants were newly diagnosed and medication-naïve. The HC group was matched for age and gender and underwent the same examinations. All subjects were informed about the scientific nature of this study and signed informed consent forms. The study received the approval of the local ethics committee.

### Neuropsychological data and clinical scales and questionnaires

Global cognitive status was assessed by the Montreal Cognitive Assessment (MOCA) (Nasreddine et al. 2005) and depression was evaluated by the Geriatric Depression Scale (GDS) (Yesavage and Sheikh 1986). All MCI-LB patients were examined by the Unified Parkinson’s Disease Rating Scale (MDS-UPDRS) III Motor Examination scores (Goetz et al. 2008), while all participants underwent the Neuropsychiatric Inventory (Cummings et al. 1994), Epworth Sleepiness Scale (Johns 1991), REM Sleep Behavior Disorder Screening Questionnaire (Stiasny-Kolster et al. 2007), and Mayo Fluctuation Scale (Ferman et al. 2004). All subjects were examined by a neuropsychological examination using a cognitive battery to assess attention (Digit Symbol Substitution and Letter-Number Sequencing (Wechsler 2006), Wechsler Adult Intelligence Scale, [WAIS-III] (Wechsler 2006), executive function (semantic and phonemic verbal fluency (Nikolai et al. 2015), Picture arrangement test as part of WAIS-III (Wechsler 2006), and visuospatial function (Judgment of Line Orientation (JLO) (Benton et al. 1994). Z-scores were calculated for each cognitive test based on normative data provided in the official test manuals, which account for age, ensuring that each participant’s score was standardized relative to an appropriate reference population. The cognitive domain scores (attention, executive functions) were then computed as the average of the z-scores of the tests within each domain. As we used only one test (JLO) for evaluation of the visuospatial domain, we refer to the test and not to the visuospatial domain z-score. To define mild cognitive impairment (MCI), we applied a threshold of −1 standard deviation (SD) below the normative mean for each test. Participants were classified as having MCI if they scored at least 1 SD below the expected score in at least two tests in one or more cognitive domains (Aarsland et al. 2010).

### MRI and ECG data acquisition

MRI data were acquired with a 3 T MRI scanner Siemens Prisma. The imaging protocol involved anatomical sequence T1 MPRAGE (iso-voxel size 0.85 mm; FoV 218 × 218 mm; TE 2.27 ms; flip angle 8°; 192 transversal slices; TR 2400 ms) and eyes-closed resting-state sequence (rs-fMRI) multi-echo multiband BOLD fMRI (multiband factor 5; TR 980 ms; flip angle 50°; TE [14.00, 34.63, 55.26] ms; FoV 200 mm; iso-voxel size 2.5 mm; 60 slices in transversal plane; 580 scans). When scanning rs-fMRI data, subjects kept their eyes closed and tried to relax and not to fall asleep.

Simultaneously to rs-fMRI data acquisition electrocardiographic data (ECG) were recorded with MR compatible BrainProducts BrainAmp (sampling frequency 5000 Hz).

### MRI data processing

Rs-fMRI data were processed with a combination of SPM12 toolbox (Penny et al. 2011) and in-house MATLAB scripts running in MATLAB 2019a with Statistics and machine learning toolbox. The preprocessing pipeline consisted of realigning, multi-echo data merging based on contrast-to-noise ratio; CNR (Poser et al. 2006; Mikl et al. 2026), spatial normalization to MNI, spatial smoothing (Gaussian kernel with FWHM 6 mm) (Preti and Van De Ville 2016; Preti and Ville 2017; Candemir 2023), motion censoring (replacing scans with FD > 1.5 mm and one preceding and two following scans using interpolation (Power et al. 2012), and regressing out nuisance regressors for white matter (first PCA component out of the thresholded probabilistic mask of white matter with a threshold > 0.9), for cerebrospinal fluid (first PCA component out of the thresholded probabilistic mask of cerebrospinal fluid with a threshold > 0.9), and 24 movement regressors (translations, rotations, their temporal derivatives, their squares, and their squares of temporal derivatives). The data were then filtered with a high pass filter (cutoff 1/128Hz); finally, the first 10 scans were excluded to eliminate artifacts related to filtering present at the beginning of the signal.

The quality of fMRI data was checked for signal dropouts with the Mask Explorer tool (Gajdoš et al. 2016) and movement-related artifacts were checked with the framewise displacement (FD) metric (Power et al. 2012). Data where FD > 0.5 mm was exceeded in more than 20% of the scans (threshold based on other studies e.g. (Power et al. 2014; Šimko et al. 2021; Schumacher et al. 2021)), were excluded from the analysis to minimize spurious motion-related correlations in the dFC results. Four HC and 6 MCI-LB subjects were excluded due to the mentioned FD criterion, and 6 HC and 1 MCI-LB were excluded due to signal dropouts and random radiological findings. Additionally, Cohen d effect sizes of mean FD were evaluated between groups and before and after motion correction procedure.

### Dynamic functional connectivity

FEC is a template-free method working with whole-brain data that computes voxel-wise eigenvector centrality to measure functional connectivity (Preti and Van De Ville 2016). Preprocessed data were analyzed based on the sliding window approach. In this study, we used ~ 60 s (61 scans) rectangular windows (Preti et al. 2017) with a step of 10 scans in the temporal domain. Shorter windows are more prone to introduce spurious fluctuations (Shakil et al. 2016). The eigenvector centralities were computed in each temporal window. MATLAB implementation of the FEC method based on the work of Preti et al.(Preti and Van De Ville 2016) was used. The computation of eigenvector centralities used the ARPACK software library implemented in MATLAB.

The representative dominant patterns (Preti and Van De Ville 2016), capturing brain states, were subsequently estimated by principal component analysis of the FECs. Mean duration and occurrence were used to quantify functional connectivity dynamics (Lamoš et al. 2021; Schumacher et al. 2021). Here the mean duration is an average duration of a continuous presence of the state in seconds and occurrence is a number of presences of the states per minute. Coverage was used as a descriptive parameter capturing percentual distribution of the states.

Elbow criterion on the sum of squared errors and second derivative of Silhouette criterion (Michel and Brunet 2019) were used to decide the optimal number of representative dominant patterns, using range 2 to 8 patterns.

Representative dominant patterns were characterized based on their local maxima and their relevance to the large-scale resting state brain networks. Hubs of the DMN, SMN, DAN, VN and the FPCN were defined with coordinates based on Gao and Lin 2012 (Gao and Lin 2012). Local maxima of a representative dominant pattern were assigned to the network if its coordinate was located within 20 mm of a network hub (Stanley et al. 2013). Moreover, Automated anatomical labelling atlas was used for characterization of the local maxima coordinates (Tzourio-Mazoyer et al. 2002).

### Correlation analyses

The occurrence and mean duration of the detected brain states entered correlation analyses with z-scores of attention and executive function domains and with a z-score of the task evaluating visuospatial functions (represented with JLO z-scores). All of these cognitive functions are known to be impaired early in the course of the disease (Scamarcia et al. 2021; Schumacher et al. 2021). Analyses were performed separately for HC and MCI-LB groups, using the Spearman correlation coefficient, *p* < 0.05, false discovery rate (FDR). Subsequently, effect of age was regressed out from FEC parameters with general linear model and partial Spearman correlation to mentioned cognitive z-scores was computed with sex and education as covariates. Cognitive z-scores already reflected age when preparing composite z-score. Non-parametric method was selected because of observed outliers in the analyzed data. Wilcoxon ranksum test was used for testing differences in continuous variables between groups. Fisher’s exact test was used for testing differences in gender. Linear regression model (LRM) was used to evaluate between-groups interactions in identified significant relations. Analyses were performed with inhouse MATLAB scripts including Statistics and machine learning toolbox.

### Analysis of vigilance

Because fluctuation of vigilance is characteristic for MCI-LB, we used power spectral density analysis of ECG as an indirect measure of vigilance. Notably, power spectral density of ECG was reported to display a high correlation with the Psychomotor Vigilance Task (Chua et al. 2012). ECG data were processed in the tool Brain Vision Analyzer. After removal of gradient artifacts, peaks of R waves were detected. Subsequent RR intervals were processed in the MATLAB toolbox HRVTool (Vollmer 2019) (v1.07) with computation of power spectral density in the 0.02 to 0.08 Hz frequency (Chua et al. 2012). Power spectral density was analyzed in a sliding window of 300 s, similarly to the work of Chua et al.(Chua et al. 2012). Mean of power spectral density across the signal was subsequently evaluated.

## Results

Altogether 7 MCI-LB and 11 HC were excluded based on the low quality of the MRI data. The final dataset thus involved 30 participants with MCI-LB (age 68.5 ± 6.0; 16 females) and 55 HC (age 67.7 ± 6.4; 33 females). Demographic and clinical data are summarized Table 1. The groups did not differ in age or gender, but they significantly differed in all three assessed cognitive domains.

**Table 1:** Demographic and clinical data of HC and MCI-LB including cognitive domains and core clinical features of MCI-LB

	HC*	MCI-LB*	*p* value
Age	55; 67.7 ± 6.4 (67.3)	30; 68.5 ± 5.9 (68.8)	*p* = 0.585
Gender	55; 22 M/33 F	30; 14 M/16 F	*p* = 0.648
Education [years]	55; 16.5 ± 3.2 (17.0)	30; 14.0 ± 3.7 (13.0)	*P* = 0.001
Attention	55; 0.6 ± 0.6 (0.5)	30; −0.2 ± 0.7 (−0.2)	*p* < 0.001
Executive functions	55; 0.3 ± 0.5 (0.3)	30; −0.4 ± 0.9 (−0.5)	*p* < 0.001
Visuospatial functions	55; 0.5 ± 0.6 (0.6)	30; −0.4 ± 1.1 (−0.3)	*p* < 0.001
Montreal Cognitive Assessment (MOCA)	55; 27.5 ± 1.1 (27.0)	30; 24.1 ± 3.2 (25.0)	*p* < 0.001
MDS-UPDRS III Motor Examination scores	N/A	30; 5.9 ± 4.1 (5.0)	N/A
Neuropsychiatric Inventory	55; 0.0 ± 0.0 (0.0)	29; 0.2 ± 0.4 (0.0)	*p* = 0.002
REM Sleep Behavior Disorder Screening Questionnaire	55; 1.7 ± 1.4 (2.0)	30; 4.1 ± 2.5 (4.5)	*p* < 0.001
Mayo Fluctuation Scale	55; 0.3 ± 0.5 (0.0)	30; 0.9 ± 1.0 (1.0)	*p* < 0.001

*Number of valid observations; [mean ± standard deviation (median)]

The elbow criterion (see Supplementary Material for details) and second derivative of Silhouette criterion (see Figure S8) identified four states as optimal; their spatial patterns are visualized in Supplementary Material Figures S1-S4. The local maxima of state #1 corresponded to the hubs of the SMN (4 maxima in the bilateral postcentral gyri and in the right precentral gyrus), DAN (3 maxima in the right precuneus, left fusiform gyrus, and right middle temporal gyrus), and VN (2 maxima bilaterally in the cuneus). The local maxima of state #2 corresponded to the hubs of FPCN (12 maxima in the bilateral inferior parietal lobule, bilateral middle frontal gyrus, and bilateral superior frontal gyrus) and DMN (2 maxima in the right angular gyrus and left superior frontal gyrus, medial part). State #3 corresponded to the hubs of FPCN (10 maxima in bilateral supramarginal gyrus, bilateral insula, middle frontal gyrus right, and middle cingulum right) and SMN (supplementary motor area left). State #4 corresponded to the hubs of FPCN (3 maxima in the inferior frontal gyrus, pars orbitalis left, and precentral gyrus right), DMN (2 maxima in the superior frontal gyrus, medial part right, and precuneus left), and SMN (postcentral gyrus left). The states’ hubs are specified in Supplementary Materials Tables S2-S5. The mean time coverage of the states 1–4 were [39.2, 18.9, 20.5, 21.3] in MCI-LB and [43.2, 16.2, 18.3, 22.3] in HC, with no significant differences between both groups.

**Fig. 1:**
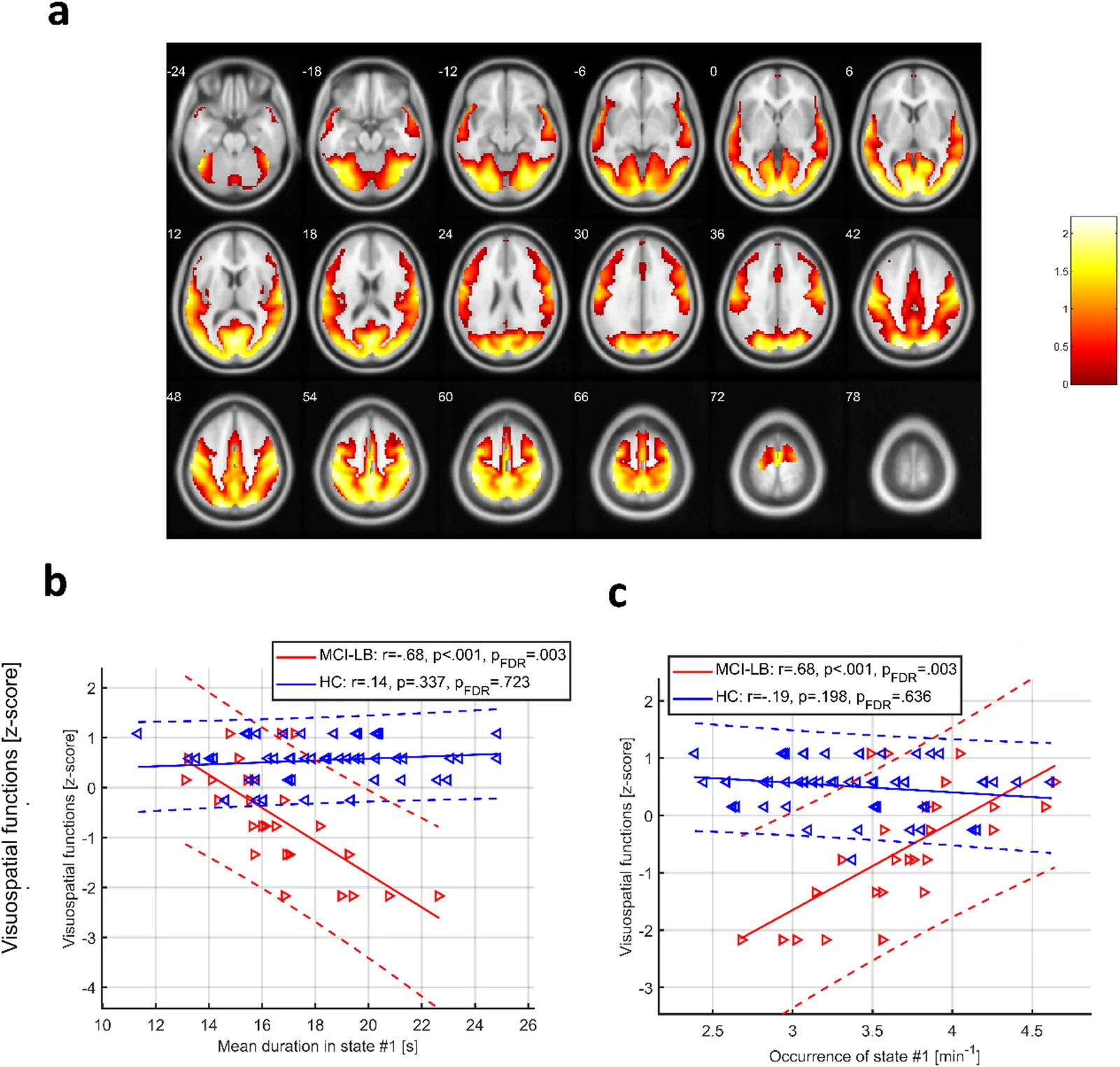
Characteristics of FEC state #1. **a** The spatial distribution of the representative dominant pattern’s eigenvector centralities. **b** Scatterplots represent the relation of visuospatial function z-scores to the mean duration of state #1 with linear trends (solid line) and 95% confidence intervals (dashed line). Correlation value is for partial correlation of Spearman R. Red is for MCI-LB and blue is for HC. **c** Scatterplots represent the relation of visuospatial function z-scores to the occurrence of state #1 with linear trends (solid line) and 95% confidence intervals (dashed line). Correlation value is for partial correlation of Spearman R. Red is for MCI-LB and blue is for HC

**Fig. 2:**
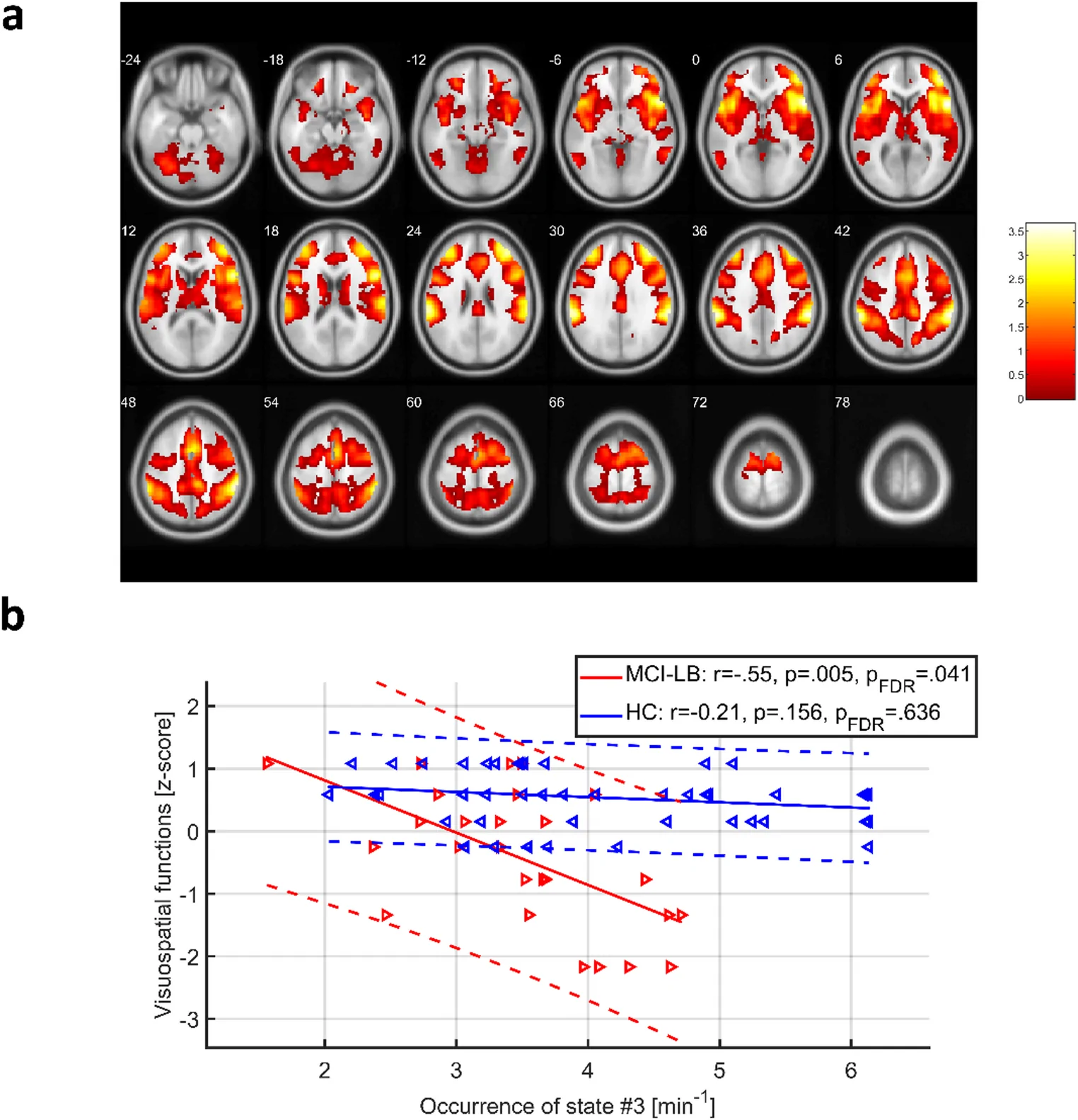
Characteristics of FEC state #3. **a** The spatial distribution of the representative dominant pattern’s eigenvector centralities. **b** Scatterplots represent the relation of visuospatial function z-scores to the occurrence of the state #3 with linear trends (solid line) and 95% confidence intervals (dashed line). Correlation value is for partial correlation of Spearman R. Red is for MCI-LB and blue is for HC

The correlation analysis revealed a significant relationship between the occurrence and of state #1 and visuospatial function z-scores (*r(24) = 0.54*, *p= 0.005*, *95% CI [0.08*,* 0.80]*, with significant LRM interaction *p* = *0.010*) and between the mean duration od state #1 and visuospatial function z-scores (*r(22) = − 0.61*, *p* = 0.002, *95% CI [−0.83*,*−0.14]*) in the MCI-LB group. The LRM interaction between MCI-LB and HC was significant in both cases with *p* = *0.010*. After controlling the analysis to the effect of age, sex and education significant relationship between the occurrence of state #1 and visuospatial function z-scores (*r(22) = 0.68*, *p = 0.003*,* 95% CI [0.23*,* 0.89]*) and between the mean duration of state #1 and visuospatial function z-scores *r(22) = − 0.68*, *p = 0.003*, *95% CI [−0.89*,* −0.24]*) remained, see Fig. 1. The LRM interaction between MCI-LB and HC was significant in both cases with *p* = *0.019* in first case and with *p* =* 0.003* in second case. We also found a statistically significant correlation between the mean duration of state #3 and visuospatial function z-scores (*r(24) = 0.54*,* p = 0.004*,* 95% CI [0.21*,* 0.73]*, with LRM interaction *p* = *0.487*) in MCI-LB (unlike in HC), but after controlling the analysis for effect of age, sex and education, this result did not survive FDR correction, with the opposite direction of correlation. However, a significant association emerged between the occurrence of state #3 and visuospatial z-scores after FDR correction (*r(22) = − 0.55*, *p = 0.041*,* 95% CI [−0.83*,* −0.15]*, with non-significant LRM interaction *p* = *0.504*), see Fig. 2. All results of the correlation analyses are presented in Supplementary Material Table S7 and Table S10. To assess whether results are related to education as a proxy of cognitive reserve/brain resilience, we further divided the MCI-LB group according to the education length mean. Notably, results remained significant in the MCI-LB individuals with lower education while they lost significance in the higher education subgroup. For detailed results, see Suppl. Material., section Effect of education.

The HC and MCI-LB groups did not differ in any of our dFC parameters of interest (Wilcoxon rank sum test, p_FDR_<0.05); all dFC parameters and p-values of between-group differences are presented in Supplementary Material Table S6.

The analysis of power spectral density in 0.02 to 0.08 Hz of RR intervals revealed no significant differences in mean power spectral density (Wilcoxon rank sum *p* = 0.694, Cohen d = 0.109) between MCI-LB and HC group. The power spectral density values are depicted in Table S9.

The effect size of movement correction on mean FD is negligible within both groups (HC_Cohen d_ = 0.016 and in MCI-LB _Cohen d_ = 0.009) and the difference between both groups in mean FD has also very small effect size (Cohen d < 0.2). Details of this comparison are depicted in Supplementary Material Table S8.

## Discussion

In the present study, we report that dynamics in fluctuations of BOLD fMRI capture variations in cognitive performance in drug-naïve MCI-LB. It was shown that data-driven representative dominant patterns of the FEC method can represent the dynamics of large-scale RSN (Preti and Van De Ville 2016). In MCI-LB, unlike in HC, FEC revealed increased temporal engagement of the state characterized by positive between-network connectivity of the DAN, SMN, and VN in relation to visuospatial performance, such that the domain z-scores were positively correlated with the state occurrence and negatively correlated with its mean duration. Interaction between MCI-LB and HC group was significant in both dFC parameters. Shorter dwell time and a more frequent state occurrence can be interpreted as a measure of (enhanced) temporal between-network flexibility (10, 11), here of the brain networks primarily engaged in LBD neurodegeneration (8), and underlying visuospatial processing control (Garcia-Diaz et al. 2018). The DAN primarily engages bilateral frontal eye fields, intraparietal sulci, and motion-sensitive middle temporal areas subserving visuospatial function, eye movements, and hand-eye coordination (Corbetta and Shulman 2002). The DAN regions involved in the dFC state #1 included the right precuneus, left fusiform gyrus, and right middle temporal gyrus. The middle temporal area and precuneus have also been identified as components of the ventral and dorsal visual streams, respectively. The dorsal visual stream has been characterized as the ‘where’ pathway, responsible for spatial attention, whereas the ventral visual stream has been identified as the ‘what’ pathway, associated with object recognition, both located particularly in the right hemisphere (Kravitz et al. 2011). The left fusiform gyrus has been implicated in visual mental imagery (Spagna et al. 2021). All the hubs of the DAN, SMN, and VN engaged in this dFC state have been reported as structural brain correlates of impaired visuospatial processing in PD subjects (Garcia-Diaz et al. 2018). We may therefore speculate that the level of network flexibility of the three major task-positive networks may facilitate visuospatial processing in early stages of LBD. Similar to our results, Asendorf et al.(Asendorf et al. 2024) showed that specific high network dynamics are relevant in motor control in cognitively unimpaired Parkinson’s disease (PD) patients and associated with dopamine transporter availability in the striatum. Indeed, visuospatial attention and processing control in LBD is related to various neurotransmitters far beyond the dopaminergic system (Kanel et al. 2020; Ophey et al. 2023). Regarding cognitive functions, Nomi et al.(Nomi et al. 2017) demonstrated that the frequency of specific brain-state occurrences is important for executive function tasks in healthy individuals. This was particularly true for configurations enabling cognitive flexibility as compared to states associated with low arousal. In the same vein, Kim et al.(Kim et al. 2017) demonstrated that interconnected between-network state occurrence encompassing the central executive network, VN, and SMN was increased in PD patients. The authors interpreted this result as a potential compensatory mechanism of intrinsic brain networks resulting in stronger synchrony. The current results can also be interpreted as increased adaptive network integration which was positively associated with a better cognitive performance. Loss of adaptive integration was previously described in fully blown LBD using dynamic EEG microstate measures in relation to fMRI dynamic connectivity magnitude between basal ganglia and cortical networks (Schumacher et al. 2019b).

On the other hand, visuospatial functions in MCI-LB were negatively correlated with occurrence of the state representing mostly within-network connectivity of the FPCN hub nodes. This result was mirrored by positive correlation between the cognitive outcome and the state dwell time, although the latter result was not significant when age, sex and education were regressed out. In other words, decreased occurrence (in this case a measure of temporal stability) of the primarily intra-network connectivity state served as another mechanism to facilitate cognition. The frontoparietal control system includes the anterior prefrontal, dorsolateral prefrontal, dorsomedial superior frontal/anterior cingulate, anterior inferior parietal lobule, and anterior insular cortex. Our dFC state #3 included particularly the anterior inferior parietal lobule, known to be involved in control of spatial attention (Sapir et al. 2005), and the insula and anterior cingulate, engaged in conflict monitoring, information integration, and response selection (Christopher et al. 2015).

Increased dwell time in the state dominated by within-network connectivity is usually regarded as network segregation, and as such it has been repeatedly related to impaired clinical symptoms of PD (Kim et al. 2017; Fiorenzato et al. 2019). However, the FPCN has a unique role in facilitating switching between the DAN and the “task-negative” DMN, thus supporting on-demand brain network dynamics and guiding cognitive processing by integrating information from the external environment with stored internal representations (Vincent et al. 2008; Cascone et al. 2021). Notably, in the study of (Firbank et al. 2024), patients with advanced LBD/PD-D group with hallucinations showed reduced connectivity of the network hubs involving ACC, bilateral anterior insula and parietal regions, i.e. the regions involved in our FPCN. This was related to worse visuospatial performance. In the current study, prodromal LBD subjects reveal increased temporal stability and network segregation of the FPCN, which was was related to better visuospatial performance.

A limitation of our study is a smaller sample size in the MCI-LB group, which could have resulted in lower statistical power for detecting between-group differences in the temporal parameters of the studied states. Another limitation is representation of visuospatial functions by a single JLO test. Additionally, the MCI-LB group was characterized clinically while no suggestive biomarkers of dementia with Lewy bodies were used such as dopaminergic imaging or polysomnography. Of note, dopaminergic imaging has low sensitivity in prodromal DLB (McKeith et al. 2020) and neither polysomnography nor α-synuclein examination using seed amplification assay from CSF or skin biopsy is broadly available in our country.

As the paradigm of the rs-fMRI measurement involved eyes closed condition, we used power spectral density of RR intervals as an indirect measure of clinical vigilance. We did not observe any differences between both groups and therefore we do not assume that the observed FEC results in the MCI-LB group were caused by the decreased levels of vigilance during MRI data acquisition. As power spectral density of RR intervals is an indirect measure, the influence of fluctuations in vigilance cannot be entirely excluded.

Taken together, our findings indicate that MCI-LB subjects engage distinct brain networks related to visuospatial processing control. Due to positive correlations with cognitive z-scores, this could be interpreted as a compensatory mechanism that seems to lead to visuospatial function facilitation in LBD subjects in the early phase of the disease, while the networks dynamics may change over time as the disease progresses to fully blown LBD. In this regards, only longitudinal studies may shed further light on this hypothesis. While compensation refers to adaptive processes in the brain that dynamically stabilize task performance despite the presence of pathology (Cabeza et al. 2018), we have to acknowledge that interpretation of the current results as “compensatory” is very speculative and much larger sample sizes would be needed and ideally task-based fMRI or an interventional condition would have to be employed to further support this notion. Based on our study design we were not able to relate the identified adaptive changes with the degree of brain pathology (e.g. demonstrating the magnitude nigrostriatal loss or degeneration of cholinergic tracts or nucleus basalis of Meynert). In fact, our results could be also related to brain resilience which refers to a cumulative capacity of neuronal resources that enables individuals to sustain function despite progressive pathology. Unlike brain compensation, this capacity develops as a consequence of lifestyle-related exposures such as e.g. education. To further address to which extend the (positive) relationship between dynamic connectivity of the states 1 and 3 and cognitive outcomes of interest is dependent on brain resilience, we divided the MCI-LB group according to their median length of education. We found that the results remained significant only in the subgroup of MCI-LB subjects with lower length of education while the correlations with cognitive outcomes were lost in the subgroup with higher length of education. In other words, the relationship between the engagement of specific dynamic states and cognitive outcomes of interest was “buffered” by cognitive reserve. Therefore, it seems plausible to state that the degree of dynamic flexibility and stability of distinct functional connectivity states at rest is (positively) associated with cognitive performance in MCI-LB and it is highly dependent on the brain resilience.

In conclusion, using dFC analysis, we were able to identify two mechanisms that were related to visuospatial variability in MCI-LB, including the network flexibility and adaptive integration of the networks involved in visuospatial function processing, and the network stability and segregation of the network particularly engaged in switching between task-positive and task-negative brain networks.

## Supplementary Information

Below is the link to the electronic supplementary material.

Supplementary Material 1

## Data Availability

The data supporting the findings of this study are not openly available. Anonymized data are, however, available from the corresponding author upon request.
