# Supplementary material for: Dynamic functional connectivity is related to cognitive performance of prodromal Lewy body dementia
Source: J Neural Transm (Vienna). 2026 Mar 31;133(9):2359–69. doi: 10.1007/s00702-026-03148-6 (PMC13687190; doi:10.1007/s00702-026-03148-6)
Supplement: Supplementary file 1 [file 702_2026_3148_MOESM1_ESM.docx]

# Supplementary Materials

## Methods

### Assessment of differences between groups in age and gender

We evaluated the differences between groups in age in terms of the mean and standard deviation of the groups. The effect size of the differences between groups in age measured with Cohen’s d is d = 0.126, and the ratio of standard deviations (HC to MCI-LB) is 1.144. As shown by Kover et al. (2013) (Kover and Atwood 2013), groups with an effect size of the differences in means with d<0.13 and a ratio of standard deviation < 1.2 can be considered as matched (Kover and Atwood 2013).

To assess differences in gender, we used Fisher’s exact test. Here with p = 0.648, we accept the null hypothesis of no statistically significant difference between groups.

### Identification of multivariate outliers

We decided to exclude multivariate outlying values to reduce the effect of outliers on the correlation analysis. For this purpose, we use the algorithm for detection of outliers from a normal multivariate sample, implemented in MATLAB as moutlier1 (<https://www.mathworks.com/matlabcentral/fileexchange/12252-moutlier1>). The calculation of the outlier is based on the Wilks' method (Wilks 1963). The algorithm uses a test with critical threshold alpha to decide about outlier, we used threshold 0.01.

### Computation of representative dominant patterns from Preti 2016

To provide more detailed view on computation of FEC representative dominant patterns in our work, we are attaching overview of the algorithm explained in full detail in (Preti and Van De Ville 2016). Obtained representative dominant pattern can be interpret as eigenconnectivities, i.e. connectivity patterns with coherent dynamics identified through principal component analysis (PCA) applied to dynamic functional connectivity (FC) data. For more detailed description please see (Leonardi et al. 2013).

*Nv* – number of volumes

*T* – number of timepoints

*X* = *Nv × T* - fMRI data matrix

*w* – window length

*s* – window step

1. fMRI matrices are split to *Xi* matrices of each window and separately analyzed
2. Eigenvector centrality is computed *Ci=XiXi^T^*, representing dominant brain pattern in particular window
3. Static eigenvector centrality is computed *C=XX^T^* and subsequently subtracted from every *Ci* to remove effect of static connectivity
4. Eigenvector decomposition is performed to get eigenvector centralities $v_{i,1}^{'}$

$$\left( C_{i}-C \right)v_{i,1}^{'}= \lambda_{i,1}^{'}v_{i,1}^{'}$$

5. In matrix *E* are aggregated eigenvector centralities across time windows $v_{i,1}^{'}$
6. PCA decomposition on *E* is performed

$$E= U_{E}S_{E}V_{E}^{T}$$

7. Here *U_E_* represent resting state brain dominant patterns, characterizing the brain at voxel level as eigenmaps.
8. Based on Pearson correlation, each time window is associated with one of the resulting representative dominant patterns. Subsequently, state vector is obtained.
9. Occurrence (number of presences of the states per minute) and mean duration (an average duration of a continuous presence of the state in seconds) of each state is computed based on state vector.

### Multi-echo preprocessing workflow

To include more details on multi-echo preprocessing workflow, we are attaching scheme of pipeline used in our work. We did not use ICA-based multi-echo denoising. Instead, we merged echoes based on contrast to noise ratio, similarly to (Mikl et al. 2026). Our pipeline consisted of these steps:

1. Spatial realignment
2. tSNR calculation (the mean signal intensity over time divided by its standard deviation)
3. CNR calculation as tSNR * TE, where TE is echo time
4. Weighted sum of realigned echo-related fMRI (echo 1 to echo 3)
5. Spatial normalization
6. Spatial smoothing (6mm FWHM)
7. Motion censoring
8. Regressing out nuisance regressors
9. High pass filtering (cutoff 1/128Hz)
10. First 10 scans excluding

### Effect of education

Due to significant differences in education between MCI-LB and HC groups and due to partial effect of using education as covariate on significance of the results we have performed a post-hoc investigation. We have divided MCI-LB group into low-education MCI-LB (le MCI-LB, N=12) and high-education MCI-LB (he MCI-LB, N=18) based on a median split. We regressed out with general linear model the effect of age from FEC parameters and subsequently run the correlation analysis with partial Spearman correlation adjusted for sex on FEC parameters and z-scores for Visuospatial, Attentional and Executive functions z-scores.

## Results

### Effect of education

In le MCI-LB group, we have observed similar significant FDR corrected results to the whole group; between the occurrence and the mean duration of state #1 and visuospatial function z-scores (*r(8) = .79, p = .032, 95% CI [0.08, 0.99]* and *r(9) = -.83, p = .032, 95% CI* *[-0.98, -0.41]*, respectively) and occurrence of state #3 and visuospatial z-scores (*r(8) = -.82, p = .032, 95% CI [-0.98, -0.41]*). We did not observe any significant results in the he MCI-LB group.

### Effect of global signal regression

Global signal regression was not performed during the preprocessing of the fMRI data. We have recalculated the fast eigenvector centrality (FEC) analysis with global signal regression to evaluate the effect of this preprocessing step. Resulting states remained clearly identifiable and almost identical with R^2^>98.8%; particularly State 1 R^2^=99.4%, State 2 R^2^=99.5%, State 3 R^2^=99.3%, State 4 R^2^= 98.8%. Although global signal regression is often considered as controversial step because of possible introducing artificial anticorrelations in the data (Weissenbacher et al. 2009), fast eigenvector centrality appears relatively robust and unaffected with this preprocessing step.

**Figure S1** Slice view of state #1 (FEC representative dominant pattern 1). The spatial distribution map displays positive eigenvector centrality values without thresholding

**Figure S2** Slice view of state #2 (FEC representative dominant pattern 2). The spatial distribution map displays positive eigenvector centrality values without thresholding

**Figure S3** Slice view of state #3 (FEC representative dominant pattern 3). The spatial distribution map displays positive eigenvector centrality values without thresholding

**Figure S4** Slice view of state #4 (FEC representative dominant pattern 4). The spatial distribution map displays positive eigenvector centrality values without thresholding

**Figure S5** Second derivative of the Silhouette criterion. The maximum suggests an optimal configuration of 4 FEC representative dominant pattern.

**Table S1** The cognitive tests and other standardized questionnaires and scales.

|  | HC* | MCI-LB* | p value** |
| --- | --- | --- | --- |
| Digit Symbol Substitution (WAIS-III) | 55; 0.5 ± 0.7 (0.3) | 29; -0.1 ± 0.9 (-0.3) | p = 0.001 |
| Letter-Number Sequencing test (WAIS-III) | 55; 0.6 ± 0.7 (0.7) | 30; -0.3 ± 0.9 (-0.3) | p < 0.001 |
| Picture arrangement test (WAIS-III) | 55; -0.1 ± 0.7 (-0.3) | 30; -1.2 ± 0.8 (-1.0) | p < 0.001 |
| Verbal fluency - lexical part | 55; 0.7 ± 0.8 (1.0) | 30; 0.2 ± 1.3 (0.5) | p = 0.141 |
| Verbal fluency - semantic part | 55; 0.4 ± 1.0 (0.5) | 30; -0.1 ± 1.1 (0.0) | p = 0.052 |
| Judgment of Line Orientation (JLO) | 55; 0.5 ± 0.6 (0.6) | 30; -0.4 ± 1.1 (-0.3) | p < 0.001 |
| Epworth Sleepiness Scale | 55; 4.4 ± 3.4 (4.0) | 30; 7.9 ± 3.9 (8.0) | p < 0.001 |
| Geriatric Depression Scale (GDS) | 55; 4.1 ± 3.3 (4.0) | 30; 8.9 ± 6.9 (8.0) | p < 0.001 |

_* Number of valid observations; [mean ± standard deviation (median)]._

_** Wilcoxon rank sum test was used to compare groups in the age and cognitive domains._

**Table S2** Coordinates of maxima in state #1 and their relation to relevant cognitive brain networks. Local maxima with a distance >20mm to the nearest network node of our interest (Gao and Lin 2012) are labelled as “other” (Stanley et al. 2013). Abbreviations: SMN - sensorimotor network, DAN – dorsal attentional network, VN – visual network

| Coordinate of local maxima in MNI [mm] | Brain network | Atlas label |
| --- | --- | --- |
| 46 -32 58 | SMN | postcentral gyrus right |
| 51 -27 53 | SMN | postcentral gyrus right |
| -45 -35 58 | SMN | postcentral gyrus left |
| 46 -10 55 | SMN | precentral gyrus right |
| 21 -75 48 | DAN | precuneus right |
| -47 -62 -18 | DAN | fusiform gyrus left |
| 51 -65 8 | DAN | middle temporal gyrus right |
| 1 -75 13 | VN | cuneus, occipital lobe right |
| -7 -85 3 | VN | cuneus, occipital lobe left |
| 13 -77 45 | other | precuneus right |
| -15 -77 45 | other | precuneus left |
| 1 -37 55 | other | paracentral lobule right |
| -2 -45 58 | other | paracentral lobule left |

**Table S3** Coordinates of maxima in state #2 and their relation to relevant cognitive brain networks. Local maxima with a distance >20mm to the nearest network node of our interest (Gao and Lin 2012) are labelled as “other” (Stanley et al. 2013). Abbreviations: FPCN – frontoparietal control network, DMN – default mode network

| Coordinate of local maxima in MNI [mm] | Brain network | Atlas label |
| --- | --- | --- |
| -47 -57 43 | FPCN | inferior parietal lobule left |
| 51 -55 45 | FPCN | inferior parietal lobule right |
| 46 21 45 | FPCN | middle frontal gyrus right |
| 38 13 55 | FPCN | middle frontal gyrus right |
| 28 61 5 | FPCN | superior frontal gyrus right |
| -40 23 45 | FPCN | middle frontal gyrus left |
| -2 46 23 | FPCN | superior frontal gyrus, medial part left |
| -2 38 35 | FPCN | superior frontal gyrus, medial part left |
| 1 28 45 | FPCN | superior frontal gyrus, medial part left |
| -37 51 10 | FPCN | middle frontal gyrus left |
| -27 56 18 | FPCN | middle frontal gyrus left |
| -20 61 18 | FPCN | superior frontal gyrus left |
| 46 -65 40 | DMN | angular gyrus right |
| 1 58 5 | DMN | superior frontal gyrus, medial part left |
| 3 31 60 | other | superior frontal gyrus, medial part right |
| 3 43 53 | other | superior frontal gyrus right |
| 6 23 65 | other | supplementary motor area right |
| 3 -72 43 | other | precuneus left |
| 1 -52 43 | other | precuneus left |
| 1 -42 45 | other | middle cingulum left |
| -27 -72 -25 | other | lobule VI of cerebellar hemisphere left |
| -40 -75 -30 | other | crus I of cerebellar hemisphere left |
| -15 -82 -25 | other | crus I of cerebellar hemisphere left |
| 26 28 55 | other | superior frontal gyrus right |
| 36 -75 -25 | other | cerebral crus right |
| 43 -70 -30 | other | cerebral crus right |
| 13 -82 -23 | other | crus I of cerebellar hemisphere right |
| -25 21 58 | other | middle frontal gyrus left |
| -32 26 50 | other | middle frontal gyrus left |
| 66 -20 -18 | other | middle temporal gyrus right |
| 66 -27 -13 | other | middle temporal gyrus right |
| -62 -27 -13 | other | middle temporal gyrus left |
| 1 -30 40 | other | middle cingulum left |

**Table S4** Coordinates of maxima in state #3 and their relation to relevant cognitive brain networks. Local maxima with a distance >20mm to the nearest network node of our interest (Gao and Lin 2012) are labelled as “other” (Stanley et al. 2013). Abbreviations: FPCN – frontoparietal control network, SMN – sensorimotor network

| Coordinate of local maxima in MNI [mm] | Brain network | Atlas label |
| --- | --- | --- |
| 58 -37 40 | FPCN | supramarginal gyrus right |
| 36 13 8 | FPCN | insula right |
| 41 13 0 | FPCN | insula left |
| -60 -37 38 | FPCN | supramarginal gyrus left |
| -62 -32 28 | FPCN | supramarginal gyrus left |
| 38 43 28 | FPCN | middle frontal gyrus right |
| 46 43 5 | FPCN | middle frontal gyrus right |
| -32 18 8 | FPCN | insula left |
| -37 13 0 | FPCN | insula left |
| 3 18 33 | FPCN | middle cingulum right |
| 3 11 48 | SMN | supplementary motor area left |
| 63 -35 30 | other | supramarginal gyrus right |
| 53 16 0 | other | inferior frontal gyrus, opercular part right |
| -37 41 28 | other | middle frontal gyrus left |
| -52 8 3 | other | rolandic operculum left |
| 11 -35 45 | other | middle cingulum right |
| 18 6 70 | other | superior frontal gyrus right |

**Table S5** Coordinates of maxima in state #4 and their relation to relevant cognitive brain networks. Local maxima with a distance >20mm to the nearest network node of our interest (Gao and Lin 2012) are labelled as “other” (Stanley et al. 2013). Abbreviations: FPCN – frontoparietal control network, DMN – default mode network, SMN – sensorimotor network

| Coordinate of local maxima in MNI [mm] | Brain network | Atlas label |
| --- | --- | --- |
| -45 33 -10 | FPCN | inferior frontal gyrus, pars orbitalis left |
| -42 23 -10 | FPCN | inferior frontal gyrus, pars orbitalis left |
| 56 -2 40 | FPCN | precentral gyrus right |
| 6 58 10 | DMN | superior frontal gyrus, medial part right |
| 1 -60 30 | DMN | precuneus left |
| -42 -17 38 | SMN | postcentral gyrus left |
| -5 51 18 | other | superior frontal gyrus, medial part left |
| 6 48 43 | other | superior frontal gyrus, medial part right |
| 26 -30 70 | other | precentral gyrus right |
| -45 16 -28 | other | temporal pole, middle temporal gyrus left |
| 36 21 -33 | other | temporal pole, superior temporal gyrus right |

**Table S6** Group comparisons of dFC parameters: mean duration [s] and occurrence [min^-1^] for all brain states in FEC. Values are mean ± standard deviation (median). P-values are for Wilcoxon rank sum test. Effect sizes are characterized with Cohen d

| DFC parameter | MCI-LB | HC | p-value | Cohen d |
| --- | --- | --- | --- | --- |
| mean duration in state #1 | 17.807 ± 4.050 (16.622) | 18.223 ± 3.119 (17.763) | 0.194 | -0.119 |
| mean duration in state #2 | 15.659 ± 3.597 (14.700) | 15.484 ± 4.695 (15.400) | 0.707 | 0.040 |
| mean duration in state #3 | 17.880 ± 5.635 (16.975) | 16.014 ± 4.810 (16.333) | 0.145 | 0.361 |
| mean duration in state #4 | 18.364 ± 5.760 (17.701) | 15.508 ± 3.190 (15.517) | 0.022 | 0.666 |
| occurrence state #1 | 3.506 ± 0.649 (3.610) | 3.390 ± 0.591 (3.378) | 0.194 | 0.189 |
| occurrence state #2 | 4.036 ± 0.951 (4.082) | 4.224 ± 1.239 (3.896) | 0.707 | -0.163 |
| occurrence state #3 | 3.613 ± 0.927 (3.535) | 4.085 ± 1.204 (3.673) | 0.145 | -0.420 |
| occurrence state #4 | 3.604 ± 1.185 (3.390) | 4.032 ± 0.842 (3.867) | 0.022 | -0.436 |

**Table S7** Spearman correlations of FEC parameters for all states and z-scores for assessed cognitive outcome. FDR correction was performed separately for MCI-LB and HC group

| DFC parameter – cognitive score pair | MCI-LB | | | | HC | | | |
| --- | --- | --- | --- | --- | --- | --- | --- | --- |
|  | r | p-value | p_FDR_ | df | r | p-value | p_FDR_ | df |
| Mean duration in state #1 and Executive functions | -0.436 | 0.030 | 0.103 | 23 | 0.192 | 0.164 | 0.437 | 52 |
| Mean duration in state #1 and Visuospatial functions | -0.606 | 0.002 | 0.040 | 22 | 0.262 | 0.063 | 0.437 | 49 |
| Mean duration in state #1 and Attention | -0.288 | 0.153 | 0.316 | 24 | -0.106 | 0.441 | 0.588 | 53 |
| Mean duration in state #2 and Executive functions | 0.009 | 0.964 | 0.964 | 27 | 0.157 | 0.298 | 0.447 | 44 |
| Mean duration in state #2 and Visuospatial functions | 0.208 | 0.280 | 0.480 | 27 | 0.167 | 0.273 | 0.437 | 43 |
| Mean duration in state #2 and Attention | 0.156 | 0.418 | 0.590 | 27 | 0.175 | 0.244 | 0.437 | 44 |
| Mean duration in state #3 and Executive functions | 0.291 | 0.126 | 0.302 | 27 | 0.080 | 0.567 | 0.680 | 51 |
| Mean duration in state #3 and Visuospatial functions | 0.542 | 0.004 | 0.040 | 24 | 0.249 | 0.081 | 0.437 | 48 |
| Mean duration in state #3 and Attention | 0.122 | 0.528 | 0.678 | 27 | -0.018 | 0.898 | 0.937 | 51 |
| Mean duration in state #4 and Executive functions | 0.122 | 0.537 | 0.678 | 26 | 0.125 | 0.374 | 0.528 | 51 |
| Mean duration in state #4 and Visuospatial functions | -0.082 | 0.696 | 0.795 | 23 | 0.214 | 0.140 | 0.437 | 47 |
| Mean duration in state #4 and Attention | 0.477 | 0.019 | 0.084 | 22 | -0.054 | 0.698 | 0.798 | 51 |
| Occurrence state #1 and Executive functions | 0.420 | 0.021 | 0.084 | 28 | -0.192 | 0.164 | 0.437 | 52 |
| Occurrence state #1 and Visuospatial functions | 0.538 | 0.005 | 0.040 | 24 | -0.259 | 0.064 | 0.437 | 50 |
| Occurrence state #1 and Attention | 0.213 | 0.306 | 0.490 | 23 | 0.167 | 0.227 | 0.437 | 52 |
| Occurrence state #2 and Executive functions | -0.009 | 0.964 | 0.964 | 27 | -0.170 | 0.263 | 0.437 | 43 |
| Occurrence state #2 and Visuospatial functions | -0.509 | 0.009 | 0.054 | 23 | -0.167 | 0.273 | 0.437 | 43 |
| Occurrence state #2 and Attention | -0.250 | 0.200 | 0.369 | 26 | -0.186 | 0.216 | 0.437 | 44 |
| Occurrence state #3 and Executive functions | -0.269 | 0.158 | 0.316 | 27 | -0.080 | 0.567 | 0.680 | 51 |
| Occurrence state #3 and Visuospatial functions | -0.296 | 0.112 | 0.299 | 28 | -0.249 | 0.081 | 0.437 | 48 |
| Occurrence state #3 and Attention | -0.178 | 0.365 | 0.548 | 26 | 0.018 | 0.898 | 0.937 | 51 |
| Occurrence state #4 and Executive functions | -0.037 | 0.855 | 0.933 | 25 | -0.170 | 0.228 | 0.437 | 50 |
| Occurrence state #4 and Visuospatial functions | 0.095 | 0.644 | 0.773 | 24 | -0.207 | 0.153 | 0.437 | 47 |
| Occurrence state #4 and Attention | -0.396 | 0.045 | 0.135 | 24 | -0.004 | 0.979 | 0.979 | 49 |

Abbreviations: r – Spearman correlation coefficient, df – degrees of freedom, p_FDR_ – p value after false discovery rate (FDR) correction

**Table S8** Quantification of movement in the HC and MCI-LB groups with mean framewise displacement (Power et al. 2012).

| Parameter | mean FD [mm] | mean FD after interpolation [mm] | differences in FD after vs before interpolation [mm] |
| --- | --- | --- | --- |
| HC * | 55, 0.174 ± 0.070 (0.164) | 55, 0.173 ± 0.069 (0.164) | 55, -0.001 ± 0.004 (0.000) |
| MCI LB * | 30, 0.186 ± 0.058 (0.173) | 30, 0.186 ± 0.058 (0.173) | 30, -0.001 ± 0.002 (0.000) |
| Cohen d | 0.178 | 0.191 | 0.180 |
| p-value ** | 0.341 | 0.318 | 0.341 |

_* Number of valid observations; [mean ± standard deviation (median)]. ** Wilcoxon rank sum test was used to compare groups in the mean FD. The effect size (Cohen d) for paired comparison in HC is 0.016 and in MCI-LB is 0.009_

**Table S9** Descriptive statistics of power spectral density of RR intervals in 0.02 to 0.08 Hz

| Parameter | HC* | MCI-LB* | Cohen d | p value** |
| --- | --- | --- | --- | --- |
| mean power spectral density of RR in 0.02-0.08 Hz | 53; 0.796 ± 0.368 (0.757) | 30; 0.837 ± 0.368 (0.809) | 0.109 | 0.694 |

_* Number of valid observations; [mean ± standard deviation (median)]. ** Wilcoxon rank sum test was used to compare the groups._

**Table S10** Spearman partial correlations with sex and education as covariates on of FEC parameters for all states and z-scores for assessed cognitive outcome. Age was regressed out from FEC parameters before partial correlation. FDR correction was performed separately for MCI-LB and HC group

| DFC parameter – cognitive score pair | MCI-LB | | | | HC | | | |
| --- | --- | --- | --- | --- | --- | --- | --- | --- |
|  | r | p-value | p_FDR_ | df | r | p-value | p_FDR_ | df |
| Mean duration in state #1 and Executive functions | -0.380 | 0.051 | 0.207 | 25 | 0.188 | 0.182 | 0.636 | 50 |
| Mean duration in state #1 and Visuospatial functions | -0.677 | <0.001 | 0.003 | 22 | 0.139 | 0.337 | 0.723 | 48 |
| Mean duration in state #1 and Attention | -0.171 | 0.403 | 0.537 | 24 | -0.094 | 0.505 | 0.759 | 51 |
| Mean duration in state #2 and Executive functions | 0.034 | 0.867 | 0.905 | 25 | 0.173 | 0.262 | 0.636 | 42 |
| Mean duration in state #2 and Visuospatial functions | 0.274 | 0.167 | 0.402 | 25 | 0.097 | 0.538 | 0.759 | 41 |
| Mean duration in state #2 and Attention | 0.137 | 0.504 | 0.576 | 24 | 0.172 | 0.265 | 0.636 | 42 |
| Mean duration in state #3 and Executive functions | 0.357 | 0.067 | 0.207 | 25 | 0.123 | 0.392 | 0.723 | 49 |
| Mean duration in state #3 and Visuospatial functions | 0.370 | 0.069 | 0.207 | 23 | 0.168 | 0.248 | 0.636 | 47 |
| Mean duration in state #3 and Attention | 0.002 | 0.992 | 0.992 | 22 | 0.020 | 0.888 | 0.999 | 49 |
| Mean duration in state #4 and Executive functions | 0.217 | 0.287 | 0.460 | 24 | -0.007 | 0.959 | 0.999 | 49 |
| Mean duration in state #4 and Visuospatial functions | 0.272 | 0.199 | 0.434 | 22 | 0.356 | 0.013 | 0.282 | 46 |
| Mean duration in state #4 and Attention | 0.195 | 0.350 | 0.494 | 23 | 0.000 | 0.999 | 0.999 | 47 |
| Occurrence state #1 and Executive functions | 0.391 | 0.040 | 0.207 | 26 | -0.191 | 0.175 | 0.636 | 50 |
| Occurrence state #1 and Visuospatial functions | 0.677 | <0.001 | 0.003 | 22 | -0.185 | 0.198 | 0.636 | 48 |
| Occurrence state #1 and Attention | 0.241 | 0.246 | 0.453 | 23 | 0.158 | 0.263 | 0.636 | 50 |
| Occurrence state #2 and Executive functions | -0.084 | 0.677 | 0.739 | 25 | -0.098 | 0.522 | 0.759 | 43 |
| Occurrence state #2 and Visuospatial functions | -0.386 | 0.052 | 0.207 | 24 | -0.078 | 0.619 | 0.810 | 41 |
| Occurrence state #2 and Attention | -0.223 | 0.264 | 0.453 | 25 | 0.101 | 0.531 | 0.759 | 39 |
| Occurrence state #3 and Executive functions | -0.291 | 0.141 | 0.375 | 25 | -0.124 | 0.387 | 0.723 | 49 |
| Occurrence state #3 and Visuospatial functions | -0.553 | 0.005 | 0.041 | 22 | -0.208 | 0.156 | 0.636 | 46 |
| Occurrence state #3 and Attention | -0.248 | 0.223 | 0.445 | 24 | -0.030 | 0.832 | 0.999 | 49 |
| Occurrence state #4 and Executive functions | -0.144 | 0.492 | 0.576 | 23 | 0.001 | 0.995 | 0.999 | 48 |
| Occurrence state #4 and Visuospatial functions | -0.146 | 0.497 | 0.576 | 22 | -0.330 | 0.024 | 0.282 | 45 |
| Occurrence state #4 and Attention | -0.209 | 0.316 | 0.475 | 23 | 0.067 | 0.642 | 0.810 | 48 |

Abbreviations: r – Spearman correlation coefficient, df – degrees of freedom, p_FDR_ – p value after false discovery rate (FDR) correction

## References for Supplementary Materials

Gao W, Lin W (2012) Frontal parietal control network regulates the anti-correlated default and dorsal attention networks. Hum Brain Mapp 33:192–202. https://doi.org/10.1002/hbm.21204

Kover ST, Atwood AK (2013) Establishing Equivalence: Methodological Progress in Group-matching Design and Analysis. Am J Intellect Dev Disabil 118:3–15. https://doi.org/10.1352/1944-7558-118.1.3

Leonardi N, Richiardi J, Gschwind M, et al (2013) Principal components of functional connectivity: A new approach to study dynamic brain connectivity during rest. Neuroimage 83:937–950. https://doi.org/10.1016/j.neuroimage.2013.07.019

Mikl M, Ingrová K, Gajdoš M, et al (2026) Complex multi-echo fMRI dataset: New strategies in processing of multi-echo data. Sci Data 13:320. https://doi.org/10.1038/s41597-026-06694-6

Power JD, Barnes KA, Snyder AZ, et al (2012) Spurious but systematic correlations in functional connectivity MRI networks arise from subject motion. Neuroimage 59:2142–2154. https://doi.org/10.1016/j.neuroimage.2011.10.018

Preti MG, Van De Ville D (2016) Eigenmaps of dynamic functional connectivity: Voxel-level dominant patterns through eigenvector centrality. In: 2016 IEEE 13th International Symposium on Biomedical Imaging (ISBI). IEEE, pp 988–991

Stanley ML, Moussa MN, Paolini BM, et al (2013) Defining nodes in complex brain networks. Front Comput Neurosci 7:1–14. https://doi.org/10.3389/fncom.2013.00169

Weissenbacher A, Kasess C, Gerstl F, et al (2009) Correlations and anticorrelations in resting-state functional connectivity MRI: A quantitative comparison of preprocessing strategies. Neuroimage 47:1408–1416. https://doi.org/10.1016/j.neuroimage.2009.05.005

Wilks SS (1963) Multivariate Statistical Outliers. Sankhyā: The Indian Journal of Statistics, Series A 25:407–426
